# How Does Crop Rotation Influence Soil Moisture, Mineral Nitrogen, and Nitrogen Use Efficiency?

**DOI:** 10.3389/fpls.2022.854731

**Published:** 2022-03-17

**Authors:** Rui Yang, Ke Liu, Matthew Tom Harrison, Shah Fahad, Zhuangzhi Wang, Meixue Zhou, Xiaoyan Wang

**Affiliations:** ^1^Hubei Collaborative Innovation Center for Grain Industry, Yangtze University, Jingzhou, China; ^2^Agriculture College, Yangtze University, Jingzhou, China; ^3^Tasmanian Institute of Agriculture, University of Tasmania, Burnie, TAS, Australia; ^4^Hainan Key Laboratory for Sustainable Utilization of Tropical Bioresource, College of Tropical Crops, Hainan University, Haikou, China; ^5^Department of Agronomy, The University of Haripur, Haripur, Pakistan

**Keywords:** dryland wheat, rice stubble wheat, yield, nitrogen use efficiencies, soil

## Abstract

Rice–wheat (RW) cropping systems are integral to global food security. Despite being practiced for decades, Chinese RW cropping systems often suffer from low productivity and poor nitrogen use efficiency (NUE), reflecting management approaches that are not well-contextualized to region and season. Here, we develop the best management guides for N fertilizer in RW systems that are designed to help raise the productivity, NUE, and environmental sustainability of winter wheat over the long term. 2-year field experiments were conducted with four N fertilizer rates (0, 135, 180, and 225 kg N ha^–1^), allowing contrasts of yields, soil moisture, and NUE of wheat in RW in the humid climates zones on the Jianghan Plain. We compared RW systems with soybean/maize dryland wheat (DW) systems that are similarly endemic to China: after soybean/maize is harvested, soils are often drier compared with moisture content following rice harvest. With high seasonal N application rates (180–225 kg N ha^–1^), wheat crop yields increased by 24% in RW which were greater than comparable yields of wheat in DW, mainly due to greater kernels per spike in the former. Across treatments and years, N accumulation in plant tissue and kernel dry matter of DW was higher than that in RW, although mean agronomic efficiency of nitrogen (AE_N_) and physiological efficiency of nitrogen (PE_N_) of RW systems were greater. As N application rates increased from 135 to 225 kg ha^–1^, AE_N_ and PE_N_ of DW decreased but changed little for RW. Soil ammonium N was much lower than that of nitrate N; changes in NH_4_^+^ and NO_3_^–^ as a consequence of increasing N fertilization were similar for RW and DW. We recommend that tactical application of N fertilizer continue seasonally until midgrain filling for both the DW and RW systems. At fertilization rates above 180 kg N ha^–1^, yield responses disappeared but nitrate leaching increased significantly, suggesting declining environmental sustainability above this N ceiling threshold. Collectively, this study elicits many functional and agronomic trade-offs between yields, NUE, and environmental sustainability as a function of N fertilization. Our results show that yield and NUE responses measured as part of crop rotations are both more robust and more variable when derived over multiple seasons, management conditions, and sites.

## Introduction

Wheat cropping systems endemic to the Jianghan Plain include dryland wheat (DW)–soybean- and rice paddy-dryland rotations, wheat being sown in winter and rice in summer. The Jianghan Plain belongs to the subtropical monsoon humid climate zone, and while rainfall can be spuriously distributed throughout the wheat growing season, probabilistically, rainfall is more prevalent between jointing and maturity (Wang et al., 2013; Li et al., 2021). Driven by differences in climate, soil, geography, agronomy, and other socio-cultural practices, yields of rice stubble wheat and DW systems vary widely (Yang et al., 2021; Yan et al., 2022). In view of the diversity in management systems across the Jianghan Plain, the development of practicable, productive, and environmentally responsible fertilization strategies is of utmost importance to ensuring the long-term sustainability of agri-food systems in the region.

In itself, crop fertilization with nitrogenous compounds is one of the most powerful tactical tools farmers have at their disposal for substantially increasing growth and yield (Rawnsley et al., 2019; Shah et al., 2021a). Double-cropping and high-intensity crop rotations conducted on a year-round basis (Zhao et al., 2021) can contribute to excessive nitrogen use and depletion, resulting in N mining and suboptimal yields (Kodzwa et al., 2020). On the other hand, excessive and/or poorly timed nitrogen fertilizer application results in both the lower nitrogen use efficiency (NUE) and significant N losses to the environment (Fageria and Baligar, 2005; Yousaf et al., 2016; Christie et al., 2018, 2020), including nitrous oxide emissions (a potent greenhouse gas), nitrate runoff, and leaching into groundwater (Harrison et al., 2014, 2016; Alcock et al., 2015; Shah et al., 2017). Scientific nitrogen fertilizer management advice should carefully consider soil nitrogen supply as a function of crop type and rotation, soil type, existing N levels, and organic matter (Bilotto et al., 2021), as well as climatic and whole farm management (Harrison et al., 2012a,b). Collectively, these factors determine the level of farm intensification and profitability (Ho et al., 2014; Harrison et al., 2017). Bu et al. (2019) contend that in rice-rapeseed rotations, soil nitrogen supply in the early stage of rapeseed is low; however, nitrogen supply of the cotton oil rotation soil at the later stage is greater: optimal N management approaches should thus increase toward later phases for these crop types, considering soil nitrogen supply and crop N demand. In actual production, the supply of soil nitrogen to crop nutrients is affected by climatic conditions (rainfall and temperature), soil (texture and pH), and cultivation management methods (rotation and tillage) (Harrison et al., 2009; Christie et al., 2014).

Both the seasonal rainfall distribution and the quantum of individual rainfall events have a large bearing on agronomic outcomes (Chang-Fung-Martel et al., 2017). Under humid climatic conditions, a single high rainfall event or continuous rainfall for multiple days will cause the nitrate in the soil to runoff with the water surface or leach into the deep soil layer, resulting in the loss of nitrogen (Christie et al., 2014; Rawnsley et al., 2019; Hess et al., 2020; Smith et al., 2021). Soil moisture is one of the foremost abiotic factors limiting wheat yield potential and nitrogen absorption (Harrison et al., 2011). Seasonal alternation of water and drought increases soil organic matter and soil water retention performance, although excess soil water will affect the nitrification of ammonium nitrogen in the soil (Yang et al., 2019; Smith et al., 2021). Paddy-upland rice rotations promote the transformation of nitrogen, influencing subsequent crop nutrient absorption; choice of crop rotation, therefore, affects the response of the collective system to nitrogen fertilization (Ibrahim et al., 2018; Ren et al., 2019). The actual effect of optimized nitrogen fertilizer management varies with soil and climatic conditions depending on contextual genotype by environment by management interactions (Liu et al., 2020; Shah et al., 2021b).

While much research has focused on approaches for improving wheat yield and nitrogen-use efficiency (NUE), this research has primarily focused on DW or rice stubble wheat single planting system. There are few comprehensive comparisons of the yield and NUE of the two. Therefore, this study carried out 2 years field experiment in the humid climatic of the Jianghan Plain to analyze the differences in soil moisture in the growing season of DW and RW and to analyze the difference in response of DW and RW yield, NUE, and soil nitrogen content to increasing nitrogen fertilizer.

## Materials and Methods

### Field Experimental Details

Field experiments were conducted at the experimental farm at Yangtze University (30°36′ N, 112°08′ E, 34 m asl) in Jingzhou, Hubei Province, China in 2017–2018 and 2020–2021.

**FIGURE 1:**
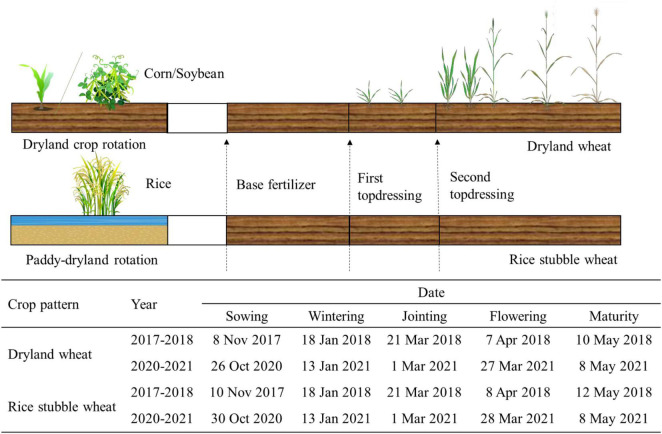
Schematic diagram of crop rotation and timing of nitrogen fertilizer application.

This experiment was conducted with two cropping rotation systems in both the cases, we only focused on the wheat within each rotation (Figure 1):

*Dryland crop rotation system*: The summer crops are dryland crops, mainly corn and soybeans, and winter wheat is DW.

*Rice-wheat rotation system*: The summer crop is rice and winter wheat is rice stubble wheat (RW). This cropping rotation system has a unique dry–wet cycle soil regime.

**FIGURE 2:**
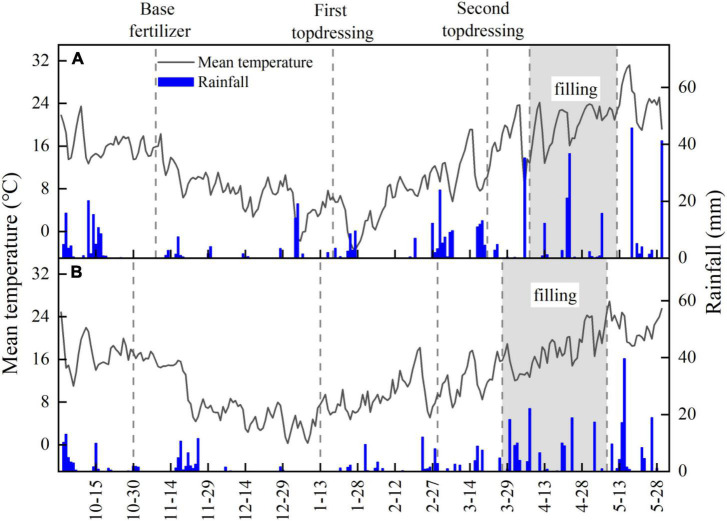
2017–2018 **(A)** and 2020–2021 **(B)** the daily mean temperature and rainfall during the whole growth period of wheat in the experimental area.

The daily mean temperature and rainfall during the wheat-growing season are shown in Figure 2. Meteorological data were collected during each growing season using an automatic weather station adjacent to experimental trials conducted here.

Before beginning the experiment, both the cropping systems had been used primarily for wheat production for more than 10 years. The tillage soil layer (0–20 cm) of the two cropping systems was as follows. Soil organic matter 11.00 g kg^–1^, available nitrogen 82.03 mg kg^–1^, available phosphorus 15.20 mg kg^–1^, available potassium 51.11 mg kg^–1^, and pH 7.8 in DW, and soil organic matter 12.37 g kg^–1^, available nitrogen 51.22 mg kg^–1^, available phosphorus 12.07 mg kg^–1^, available potassium 52.74 mg kg^–1^, and pH 7.79 in RW.

The treatments were arranged in a split-plot design with a cropping system as the main plots and N treatment as the subplots. Nitrogen fertilization treatments (timing and amount) were designed in line with local management. The four N treatments were applied as follows: 0 kg N ha^–1^ (N_0_), 135 kg N ha^–1^ (N_135_), 180 kg N ha^–1^ (N_180_), and 225 kg N ha^–1^ (N_225_), of which 180 kg N ha^–1^ is the local conventional N application rate. N fertilizer was applied at the basal, tillering, and jointing initiation stages in a ratio of 1: 1: 1, being 45 kg N ha^–1^ for the N_135_ treatment, 60 kg N ha^–1^ for the N_180_ treatment, and 75 kg N ha^–1^ for the N_225_ treatment, respectively. Each plot is repeated four times, arranged in random block groups, with a plot area of 12 m^2^ (2 m × 6 m). The experimental design and wheat growth process are shown in Figure 1. In the experiment, “Zhengmai 9023” was used as the tested variety, and the planting density was 2.25 million plants ha^–1^. N fertilizer was applied in the form of urea (46% N) that was ploughed into the soil during sowing and was spread as top dressing. Plots were supplied with P (105 kg P_2_O_5_ ha^–1^, calcium superphosphate) and K (105 kg K_2_O ha^–1^ potassium sulfate) fertilizer during the sowing period. We did not need to irrigate during the two growing seasons due to the abundant rainfall. Herbicides, pesticides, and fungicides were sprayed according to standard growing practices to avoid yield loss.

### Measurement of Yield and Yield Components

We selected mature plants from a 2 m^2^ harvest area in the middle of each plot to determine the yield and yield components. The spike number was enumerated in each plot and recorded prior to harvest. All spikes were manually harvested, threshed, and weighed to calculate grain yield. The average number of kernels per spike was enumerated from 30 spikes. One thousand random kernels from each harvested grain were weighed to calculate a 1,000-kernel weight. Grain moisture was measured using a grain analyzer (InfratecTM, Foss, Denmark). Grain yield and 1,000-kernel weight were adjusted to 13% moisture.

### Measurement of N Accumulation and Nitrogen Use Efficiency

The shoot biomass was harvested from twenty mature plants in each plot at the flowering and maturity. Samples were oven-dried at 65°C for at least 48 h until they were a constant weight. Then, pulverized with a plant grinder, followed by digestion in sulfuric acid at 400°C. Then, the N contents in the plant were measured with an automatic Kjeldahl analyzer (Kjeltec-8400, FOSS, Denmark) and the values were used to calculate the agronomic efficiency of nitrogen (AE_N_) and physiological efficiency of nitrogen (PE_N_).

*AE*_*N*_ = (Grain yield in the plot that received N applied–grain yield in the zero-N control)/(amount of N applied).

Which represents the amount of yield increased per unit N fertilizer, reflecting the economic performance of N fertilizer in terms of grain production.

*PE*_*N*_ = (Grain yield in the plot that received N applied–grain yield in the zero-N control)/(total aboveground plant N accumulation in the plot received N applied–total aboveground plant N accumulation in the zero-N control).

Which represents the yield increase per kg increase in N uptake from fertilizer. It expresses the efficiency with which the plant utilizes N taken up to produce fruit dry biomass.

### Measurement of Soil Moisture Content, Nitrate, and Ammonium Nitrogen

Soil samples were collected from each plot at the flowering (March 28, 2021), midfilling (April 14, 2021), and maturity (May 8, 2021) of wheat. Soil samples to a depth of 60 cm were obtained in triplicate by a soil auger (5-cm diameter) at 20-cm intervals. In total, 10 g of each sample was taken for determinations of nitrate (NO_3_^–^) and ammonium (NH_4_^+^), and the remaining dose was used for a gravimetric water content determination. The 10 g of the soil sample was extracted with 50 ml of 1 mol l^–1^ KCl to obtain the nitrate and ammonium concentrations in the soil. All nitrate and ammonium concentrations in various solutions were determined using A SMARTCHEN automatic intermittent chemical analyzer. The gravimetric soil moisture content of the samples was subsequently measured using the oven-drying method (105°C for 24 h).

### Statistical Analysis

Data were analyzed using a three-way (year, cropping system, and nitrogen management) ANOVA with SAS 9.2 (SAS Institute, Cary, NC, United States). Treatment means were compared using the least significant differences (LSD) at *P* < 0.05.

## Results

### Climate Conditions During the Wheat Growing Season

The daily mean temperatures for the wheat filling period were 20.4°C in 2017–2018 and 17.6°C in 2020–2021. The reduced temperature in the post-flowering period extends the length of the grain filling period in 2020–2021(Figure 2). The total rainfall for the wheat growing season was 351.1 mm in 2017–2018 and 263.8 mm in 2020–2021. In 2017–2018, the rainfall from wintering to jointing of wheat was 149.4 mm, accounting for 43% of the total rainfall during the growth period. In 2020–2021, the rainfall from the winter to the jointing of wheat was only 44.5 mm. The rainfall in this year was mainly concentrated in the filling period reached 132.6 mm, accounting for 50% of the rainfall in the entire growth period of wheat, 39% more than that in the same period in 2017–2018. Compared with 2017–2018, the frequency of rainfall in the filling period in 2020–2021 is higher. Rainy days accounted for 44% of the total filling days. The daily mean rainfall of the rainy days in the filling period was 7.0 mm, of which 57% was concentrated in the early stage of filling.

### Soil Moisture Content (%)

**FIGURE 3:**
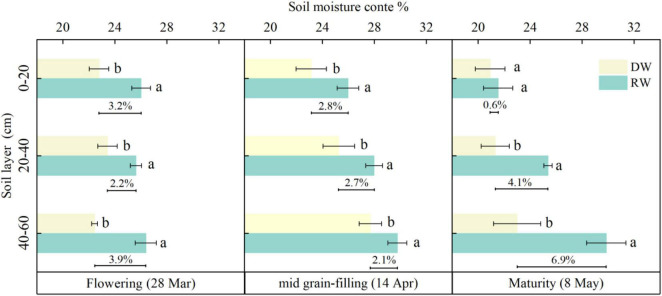
Soil moisture content in different rotation wheat seasons in 2020–2021 (0–60 cm). Different lowercase letters indicate a significant difference (*P* < 0.05) between dryland wheat vs. rice stubble wheat.

Figure 3 shows the 0–60 cm soil moisture content during the grain filling period of DW and rice stubble wheat (RW) in 2020–2021. The soil moisture content of RW was significantly (*P* < 0.05) higher than DW (except for 0–20 cm at maturity), higher 0.6–3.2% at 0–20 cm, 2.2–4.1% at 20–40 cm, and 2.1–6.9% at 40–60 cm soil depth than DW.

### Yield and Yield Components

Increasing N significantly improved grain yield in DW and RW, with significant differences between years (Table 1). Compared with N0, the yield of N supply increased by 1.2–1.4 times (RW), 0.5–0.7 times (DW) in 2017–2018; the yield increased by 2.0–2.5 times (RW), 1.6–1.7 times (DW) in 2020–2021, increased N supply has a greater effect on the increase in RW. The yield of DW was higher than that of RW, which was more pronounced in the N_0_ and N_135_ treatment in 2020–2021. When the N supply was increased from 135 to 180 kg ha^–1^, the yield increase of DW and RW will only reach significant levels in 2020–2021. When the N supply was increased from 180 to 225 kg ha^–1^, the yield increase of DW and RW was not significant in 2017–2018 and 2020–2021.

**TABLE 1:** Wheat yield and yield composition at dryland wheat (DW) and rice stubble wheat (RW).

Growing season	Crop pattern	N treatment	Grain yield (kg ha^–1^)	Spike number (10^4^ ha^–1^)	Kernels per spike	1,000-grain weight (g)
2017–2018						
	DW	N_0_	3755.2bA	316.8bA	29.7cA	40.4aA
		N_135_	5967.0aA	479.5aA	35.2bA	38.5aA
		N_180_	6418.0aA	480.5aA	38.6aA	39.1aA
		N_225_	5804.3aA	509.3aA	37.9aA	39.1aA
		**Mean**	**5486.1**	**446.5**	**35.4**	**39.3**
	RW	N_0_	1253.6bB	292.5bA	18.9bB	39.6aA
		N_135_	5808.7aA	413.3aA	37.1aA	40.0aB
		N_180_	6558.7aA	459.3aA	39.1aA	40.0aA
		N_225_	5859.8aA	437.5aB	37.8aA	40.9aA
		**Mean**	**4870.2**	**400.7**	**33.2**	**40.1**
2020–2021						
	DW	N_0_	1412.3cA	230.3cA	27.2bA	34.4aB
		N_135_	3141.4bA	293.3bA	31.7aA	39.6aA
		N_180_	3564.6aA	343.7abA	31.9aB	39.0aA
		N_225_	3652.5aA	391.3aA	34.4aB	40.7aA
		**Mean**	**2942.7**	**314.7**	**31.3**	**38.4**
	RW	N_0_	1031.4cB	182.3cB	23.2cA	39.2aA
		N_135_	2844.6bA	282.0bA	33.9bA	39.9aA
		N_180_	3479.4aA	309.0aA	36.1abA	40.0aA
		N_225_	3567.8aA	311.0aB	39.1aA	40.8aA
		**Mean**	**2730.8**	**271.1**	**33.1**	**40.0**
ANOVA						
Year (Y)			298.05**	195.06**	20.69**	1.05ns
Pattern (P)			9.95**	23.60**	0.11ns	6.72*
N fertilizer (N)			111.93**	62.20**	156.51**	3.88*
Y × P			1.73ns	0.02ns	17.99**	0.47ns
Y × N			7.95**	1.98ns	7.61**	3.51*
P × N			6.38**	1.33ns	27.28**	0.69ns
Y × P × N			4.32*	0.91ns	2.12ns	3.04*

Different lowercase letters on the same column indicate a significant difference (p < 0.05) between N treatments. Different uppercase letters on the same column indicate a significant difference (p < 0.05) between dryland wheat vs. rice stubbly wheat. *P < 0.05; **P < 0.01. ns, not significant.

The yield components of wheat were both significantly (*P* < 0.05) affected by planting patterns and N treatments (Table 1). The spike number of DW was 4–26.3% higher than that of RW under the same treatment, while RW has an advantage in the kernels per spike. Increased N supply has a difference in the increase of the spike number and kernels per spike of DW and RW. The highest increase in the spike number of DW and RW by increasing N supply by an average of 65.3 and 63.8%, respectively, over the two wheat growing seasons. The highest increase in the kernels per spike of DW was 28.3% on average, while RW could reach 87.8%. The results showed that increased N supply has a greater effect on increasing the kernels per spike in RW. Affected by the low rainfall from the tillering to the jointing of wheat and the excessive rainfall during the wheat filling in 2020–2021 (Figure 2), the spike number and kernels per spike of wheat under the same treatment in 2020–2021 was significantly lower than that in 2017–2018, and the 1,000-grain weight was not significant differences.

### N Accumulation and Nitrogen-Use Efficiency

There were significant differences in the N accumulation and NUE between DW and RW (Table 2). Under the same N supply, the N accumulation in plant tissue and kernel dry matter of DW in 2017–2018 was 9.2–168.9% and 31.2–199.4% higher than at RW. There were significant differences only under N_0_ treatment in 2020–2021. The agronomic efficiency of nitrogen (AE_N_) and physiological efficiency of nitrogen (PE_N_) at RW were significantly higher than at DW by 11.4–17.4 and 15.3–25.6 kg kg^–1^. RW was also higher than DW in 2020–2021, but the difference was not significant. The plant N accumulation and NUE of DW and RW were different in years. The N accumulation in 2017–2018 was higher than that in 2020–2021, and there was a significant difference between DW and RW in the first year. The second year also showed the same regular difference, but the difference did not reach a significant level.

**TABLE 2:** Responses of nitrogen accumulation and nitrogen use efficiency of wheat under dryland wheat (DW) and rice stubble wheat (RW) to increased nitrogen fertilizer application.

Growing season	Crop pattern	N treatment	Plant N accumulation at anthesis	Plant N accumulation at maturity	Grain N accumulation	AE_N_	PE_N_
2017–2018							
	DW	N_0_	55.1dA	77.0cA	60.4dA	—	—
		N_135_	123.9cA	164.3bA	121.4cA	16.4aB	25.0aB
		N_180_	143.6bA	207.9aA	150.4bA	14.8abB	20.1abB
		N_225_	174.7aA	218.4aA	160.4aA	9.1bB	14.5bB
		**Mean**	**124.32**	**166.92**	**123.16**	**13.43**	**19.9**
	RW	N_0_	18.4cB	32.1cB	22.5cB	—	—
		N_135_	94.4bB	144.9bB	111.2bB	33.7aA	40.3aA
		N_180_	105.7aB	156.6aB	123.1aB	29.5bA	42.7aA
		N_225_	109.0aB	147.6abB	118.2abB	20.5cA	40.1aA
		**Mean**	**81.90**	**120.30**	**93.74**	**27.90**	**41.0**
2020–2021							
	DW	N_0_	29.1cA	30.0cA	19.3bA	—	—
		N_135_	78.4bA	83.3bA	60.3bA	12.8aA	32.6aA
		N_180_	107.6aA	116.7aA	70.2aA	12.4aA	26.3bA
		N_225_	116.1aA	126.1aA	71.0aA	10.0bA	23.6bA
		**Mean**	**82.8**	**89.0**	**55.2**	**11.7**	**27.5**
	RW	N_0_	21.7dB	22.3dB	11.5dB	—	—
		N_135_	71.7cA	86.3cA	51.0cA	13.4aA	28.3aA
		N_180_	97.4bA	106.8bA	62.3bA	13.6aA	29.1aA
		N_225_	111.5aA	124.2aA	75.8aA	11.3aA	24.9aA
		**Mean**	**75.6**	**84.9**	**50.1**	**12.8**	**27.4**
ANOVA							
Year(Y)			212.71**	1087.44**	1155.96**	135.10**	6.01*
Rotation(P)			229.73**	218.31**	110.33**	114.70**	74.57**
N fertilizer(N)			677.37**	920.37**	476.27**	28.38**	7.67**
Y × P			114.95**	152.93**	55.08**	86.23**	75.16**
Y × N			8.71**	32.91**	37.87**	9.84**	0.12ns
P × N			4.99**	12.45**	2.80*	1.13ns	4.28*
Y × P × N			7.09**	8.01**	8.68**	1.78ns	0.38ns

Different lowercase letters on the same column indicate a significant difference (p < 0.05) between N treatments. Different uppercase letters on the same column indicate a significant difference (p < 0.05) between dryland wheat vs. rice stubbly wheat. *P < 0.05; **P < 0.01. ns, not significant.

Increased N supply has a significant impact on the plant N accumulation and NUE at DW and RW (Table 2). With the increase of N supply, the N accumulation in plant tissue and kernel dry matter of DW at flowering and maturity increased significantly. Compared with N_135_ treatment, the N accumulation at flowering and maturity of N_180_ treatment increased by an average of 26.6 and 23.9%, respectively, over the two wheat growing seasons, and RW increased by 33.3 and 15.9%. The corresponding N accumulation in the kernel of DW and RW increased by 20.2 and 16.4%, respectively. The increase of N_225_ treatment compared with N_180_ treatment reached a significant level only in DW in 2017–2018 and RW in 2020–2021. With the increase of N supply, AE_N_ and PE_N_ of DW decreased significantly, but AE_N_ and PE_N_ of RW had no significant difference between nitrogen treatment (except for AE_N_ in 2017–2018).

### Soil NO_3_^–^-N and NH_4_^+^-N

**FIGURE 4:**
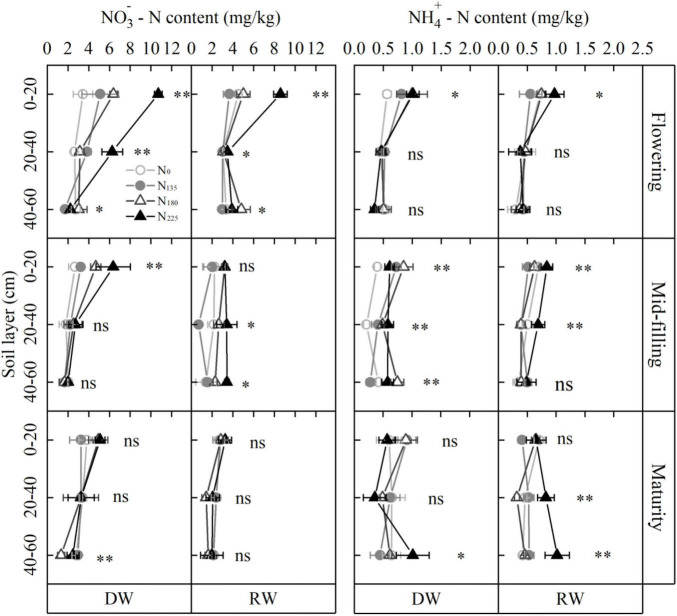
Changes in soil NO_3_^–^–N and NH_4_^+^–N content during the late growth stage of wheat under dryland wheat (DW) and rice stubble wheat (RW) in 2020–2021. *P < 0.05; **P < 0.01; ns, non-significant.

In the flowering, both DW and RW 0–20 cm soil NO_3_^–^-N increased with the increase of N supply, and both reached the maximum under N_225_ treatment (Figure 4). Which were significantly increased by 4.3–7.3 mg kg^–1^ at DW, 3.6–4.9 mg kg^–1^ at RW, compared with other treatments; But at 20–40 cm soil layer, the increase of N supply gradually reduced the soil NO_3_^–^–N content. The DW soil NO_3_^–^–N content of N_225_ treatment increased 2.4–3.6 mg kg^–1^ compared with other n treatments, while RW Only increased by 0.4–0.5 mg kg^–1^. With the deepening of the soil layer, the difference in the soil NO_3_^–^–N content at DW and RW between N treatments gradually decreased. In the middle of filling, only the 0–20 cm soil NO_3_^–^–N content at DW has significant differences between N treatments, while at RW was not significant; There was no significant difference in the NO_3_^–^–N content at DW in 20–60 cm soil between N treatments, but the N_225_ treatment significantly increased 0.8–2.7 mg kg^–1^ compared with other N treatments in RW. In the maturity, the 0–40 cm soil NO_3_^–^–N content at DW and RW had no significant difference between N treatments.

The content of NH_4_^+^–N in the soil is low, only 7.4–46.2% of the corresponding nitrate-nitrogen content (Figure 4). In the flowering, increased N supply increased the 0–20 cm soil NH_4_^+^–N content at DW and RW. Compared with other N treatments, N_225_ treatment increased by 0.02–0.45 mg kg^–1^ under DW and RW. The NH_4_^+^–N in 20–60 cm soil was not significantly different between N treatments. In the middle of filling, there were significant differences in the NH_4_^+^–N at DW in 0–60 cm soil between N treatments, while the difference in RW was only in the 0–40 cm soil layer. At maturity, there were no significant differences in NH_4_^+^–N between DW and RW in the 0–20 cm soil layer, but there were significant differences between the 40 and 60 cm soil layer.

## Discussion

This study compared the nitrogen accumulation and yield of DW and rice stubble wheat (RW) in response to different nitrogen levels. Our research results show that there were differences in nitrogen accumulation and yield of wheat between years, and the effect of nitrogen fertilizer on RW is better than DW, and RW has a higher NUE.

### Differences in Soil Moisture Content and Mineral Nitrogen Between Dryland Wheat and Rice-Wheat

The type of soil texture and the depth of the soil layer are the main factors that affect the soil moisture pattern. From the perspective of the vertical change characteristics of soil water content, the water content of the 0–60 cm soil layer increases with the increase of depth (Figure 3). Because there are more residual straws in the soil surface layer, the decomposed humus promotes the formation of soil porosity (Turmel et al., 2015), and the wheat root system is mainly distributed in the surface layer (Yang et al., 2021). The soil surface layer has a large infiltration rate, which is not conducive to the preservation of soil moisture. Therefore, the soil moisture content of the soil surface layer is likely to fluctuate with rainfall. There was a big difference in soil moisture between DW and RW. Rotation methods will change the physical properties of the soil. Long-term paddy-rice rotation promotes soil granulation. The soil texture of RW was stickier and its soil moisture content is 2.2–4.3% higher than that of DW on average (Figure 3). This may be mainly because the rice stubble wheat soil with fine texture and high field water holding capacity will more effectively store rainwater with high rainfall (Yoo et al., 1998).

The concentration of mineral nitrogen is an important index to evaluate the available nitrogen supply capacity of the soil, and the change of soil moisture is one of the most important environmental factors affecting nitrogen mineralization and nitrification. Studies have suggested that nitrification is more sensitive to water than ammonification, and the nitrification rate will be inhibited when the soil moisture is high (Schimel and Parton, 1986; Wang et al., 2021). This is mainly because the increase in rainfall results in a large soil moisture content, which is not conducive to the diffusion of oxygen in the soil. Nitrifying bacteria prefer aerobic conditions. Excessive rainfall makes clay loam soils with higher field water holding capacity more likely to form an anaerobic environment, inhibiting nitrifying bacteria and nitrification (Cameron et al., 2013). Soil available nitrogen supply was affected by soil water content, and rice stubble wheat has a greater decrease in nitrate-nitrogen content in the middle of grain filling when the soil water content was higher. Armstrong et al. (1996) found that the response of dryland sorghum to nitrogen application largely depends on the soil moisture content, and the response to nitrogen increase depends on the time of rainfall. The results of this study can also prove this point. Due to the influence of soil water content and high rainfall frequency, the nitrate-nitrogen content of the surface soil of rice stubble wheat is less responsive to increased nitrogen fertilizer application than DW. This is because of the three ways of soil nutrient loss (gas loss, leaching loss and runoff, and erosion loss), both leaching loss and runoff loss are highly susceptible to rainfall intensity. The Jianghan Plain has abundant rainfall, with a total rainfall of 132.6 mm during the wheat filling period in 2020–2021, which is a humid climate zone (Figure 2). The soil nitrogen loss pathways of rice stubble wheat and DW have a great relationship with soil texture and soil moisture. When the rainfall of the fine-textured paddy soil exceeds the infiltration amount of the soil, the field water holding capacity will reach a saturated state, and the field water is more likely to form surface runoff (Zhao et al., 2012); The rough soil has strong water conductivity, fast soil infiltration rate, and more serious leaching loss (Sugita and Nakane, 2007). We suggest that the interplay between soil water and soil nitrogen in crop rotations warrants further attention: part of this could include direct quantification of the association between soil moisture and soil nitrogen at the beginning of the cropping phase and subsequent implications for both growth and NUE.

### Differences in Nitrogen Accumulation Between Dryland Wheat and Rice-Wheat

This study adds significant novelty to the scientific literature as we compare: (1) cropping systems rotational effects (RW or soybean/corn–wheat) on soil nitrogen and crop NUE and (2) our contrast between RW and DW systems allows direct comparison of the effects of residual soil moisture on subsequent crop growth and NUE. Indeed, soil mineral nitrogen concentration is often linked with the vegetative growth of wheat. Some studies have shown that high levels of mineral N (NO_3_^–^ and NH_4_^+^) stimulate shoot biomass accumulation of fertilized treatments, neither seed yield nor seed nitrogen concentration was affected by soil mineral N availability (Voisin et al., 2002). In contrast, this study has shown that the yield and nitrogen accumulation of RW were related to nitrogen application rate and soil mineral nitrogen content at flowering (Table 2). Increasing nitrogen application significantly increased the soil mineral nitrogen content of rice stubble and wheat, and increased plant nitrogen accumulation. Compared to the RW, plant N accumulation at maturity was greater at the DW. Only when the N accumulation at DW at the flowering stage was greater than at RW, the N accumulation of at DW at maturity was greater at RW (Table 2).

Excessive rainfall at the time of fertilization may cause loss of external nitrogen sources at RW soil because the amount of fertilizer-N applied before flowering initiation was similar at the two systems. Therefore, in areas with high rainfall, RW may be more suitable for small and multiple fertilization, especially to avoid the high-frequency rainfall period (March–May). Even though Ney et al. (1997) stressed that any diagnosis using the critical nitrogen concentration was best done before seed filling. However, the more rainfall from March to May in Jianghan Plain makes the nitrogen supply in different wheat growing environments different, so we believe that it is equally important to understand the soil nitrogen supply in the later stages of wheat growth.

### Differences in Yield and Nitrogen-Use Efficiency Between Dryland Wheat and Rice-Wheat

Under low nitrogen (0–135 kg ha^–1^), the yield of DW was higher than that of RW, and under conditions of sufficient nitrogen supply, RW can also obtain the same yield as DW. The results show that the soil of DW has a higher basic nitrogen supply capacity. In this experiment, DW has a higher spike number and nitrogen accumulation at flowering. The soil of DW provides more nitrogen in the early growth period and has more tillers at wintering and spike number at harvest (Yin et al., 2018). This may be the reason for the higher yield of DW and the higher accumulation of nitrogen at flowering. Compared with DW, the conversion of paddy fields to dryland causes changes in the physical and chemical properties of the soil. Poor soil aeration has an adverse effect on the emergence of RW and the growth of the seedling stage (Nawaz et al., 2016). Therefore, the spike number at maturity of RW is less than that of DW. The yield increase effect of RW was mainly that the number of kernels per spike was increased by 71.2–84.4% (Table 1). Due to decreased shoots per unit area and alleviated competition for resources between shoots (Guo and Schnurbusch, 2015).

Prior to this study, the greater root length density is an important root trait for the crop with high grain yield and higher NUE under N-limited conditions or at lower N rates (Ju et al., 2015). Our previous results indicated that the root length density at DW was greater at RW, especially in deep soil layers. This may also be the reason why the N accumulation at DW was greater at RW (Yang et al., 2021). AE_N_ refers to the amount of yield increased per unit N fertilizer, reflecting the economic performance of N fertilizer in terms of grain production. Although the N accumulation at RW was lower than at DW, it can reach a similar yield level at DW. Therefore, RW has greater N use efficiency. Higher nitrogen accumulation level or NUE can achieve high yield.

## Conclusion

The effect of increasing nitrogen fertilizer on the yield at rice stubble wheat was greater, and the main reason for the increase was the increase in the number of kernels per spike. The nitrogen accumulation at rice stubble wheat was not as good as at DW, but it can reach the same yield level. The agronomic nitrogen efficiency and physiological use efficiency at rice stubble wheat were better than at DW. From the perspective of soil nitrogen levels, higher rainfall and higher field water holding capacity can easily lead to the loss of soil nitrogen at rice stubble wheat. Overall, based on the humid climatic conditions and different soil environmental characteristics of the Jianghan Plain, the nitrogen fertilizer application rates for both DW and rice stubble wheat should not exceed 180 kg ha^–1^.

## Data Availability Statement

The original contributions presented in the study are included in the article/supplementary material, further inquiries can be directed to the corresponding authors.

## Author Contributions

RY and XW initiated and designed the research. RY and ZW performed the experiments. MH, MZ, KL, SF, and XW revised and edited the manuscript and also provided advice on the experiments. All authors contributed to the article and approved the submitted version.

## Conflict of Interest

The authors declare that the research was conducted in the absence of any commercial or financial relationships that could be construed as a potential conflict of interest.

## Publisher’s Note

All claims expressed in this article are solely those of the authors and do not necessarily represent those of their affiliated organizations, or those of the publisher, the editors and the reviewers. Any product that may be evaluated in this article, or claim that may be made by its manufacturer, is not guaranteed or endorsed by the publisher.
